# TransPilot: mining key transcription factors by correlating binding sites with differentially expressed genes

**DOI:** 10.1093/bioadv/vbag145

**Published:** 2026-05-23

**Authors:** Huang Tinghua, Yao Min, Zhang Fanghong, Niu Siqi, Wen Jingchun, Wang Jianwu, Yang Ye, Gao Xuejun

**Affiliations:** College of Animal Science and Technology, Yangtze University, Jingzhou 434025, China; College of Animal Science and Technology, Yangtze University, Jingzhou 434025, China; College of Animal Science and Technology, Yangtze University, Jingzhou 434025, China; College of Animal Science and Technology, Yangtze University, Jingzhou 434025, China; College of Animal Science and Technology, Yangtze University, Jingzhou 434025, China; College of Agriculture, Yangtze University, Jingzhou 434025, China; College of Animal Science and Technology, Yangtze University, Jingzhou 434025, China; College of Animal Science and Technology, Yangtze University, Jingzhou 434025, China

## Abstract

**Motivation:**

Current software packages to mine key transcription factors (TFs) regulating the differentially expressed genes (DEGs) have not been satisfactorily utilized.

**Results:**

Here, we present TransPilot, a web server that identifies key TFs from transcriptome data via weighted Kendall’s tau rank correlation of ranked and directed TF-target sets against DEG lists. TransPilot employed an artificial neural network (ANN), which was trained using features derived from a log-likelihood ratio (LLR) array model, to identify transcription factor binding sites (TFBSs). The TF-target sets were ranked based on the ANN scores, which represent the binding potentials of the TFBSs. An imputation method was introduced to fill in the LLRs for genes that lack TFBS in their promoters. Most importantly, the direction for each ranked TF-target pair was annotated as positively or negatively regulated based on the correlation coefficient of their expression profiles in a reference transcriptome dataset. The key TFs were identified from transcriptome data by testing the correlation between the order of genes in the TF-target sets and the corresponding order in the DEG list. The analysis emphasizes end-ranked genes with large weights and de-emphasizes middle-ranked genes with small weights. The server was benchmarked on a transcriptome dataset derived from a macrophage polarization experiment.

**Availability and implementation:**

The data, code, and results utilized in this study can be accessed at http://www.thua45.cn/transpilot.

## 1 Introduction

Transcription factors (TFs) bind to specific sequence motifs in the promoters of genes, regulating the transcription process from template DNA to mRNA. The prediction of the locations of these sequence motifs, known as transcription factor binding sites (TFBS), throughout the genome is often referred to as the “motif scan” task (Ramsey *et al.* 2008). In recent decades, a plethora of computational software packages have been developed to address this challenge. These packages typically align the position weight matrix (PWM), which describes the binding preferences of TFs, with the promoter sequence of genes. Five notable software packages have been documented since 2010: TFM_EXPLORER (Tonon *et al.* 2010), SWAN (Kim *et al.* 2010), FIMO (Grant *et al.* 2011), PWMScan (Ambrosini *et al.* 2018), and Motif_scraper (Roberson 2018). In a recent study, a computational TFBS prediction framework was proposed, and mixed Student’s *t*-test statistics were established. This methodological advancement was then utilized to develop GRIT, a TFBS prediction software (Huang *et al.* 2022).

The key TFs that regulate the differentially expressed genes can be identified through first construction of TF-target sets (comprised of genes targeted by a specific TF) and a DEG list (containing differentially expressed genes), followed by the implementation of enrichment or correlation analysis on these lists. Statistical methods such as Fisher’s exact test and its variants (Lachmann *et al.* 2010, Keenan *et al.* 2019, Magnusson and Lubovac-Pilav 2021, Yao *et al.* 2021) and Kolmogorov–Smirnov statistics and its variants (Subramanian *et al.* 2005, Maleki *et al.* 2020) provided robust options for enrichment analysis. Widely used software implementing these statistical methods includes GOStats (Falcon and Gentleman 2007), ClusterProfiler (Yu *et al.* 2012), GSEA (Subramanian *et al.* 2005), WebGestalt (Wang *et al.* 2017), and others (Maleki *et al.* 2020). However, these software packages were unable to make use of the rank information of genes in the TF-target sets. In recent advancement, an algorithm was proposed to first construct the ranked TF-target set and ranked DEG list, followed by performing a weighted Kendall’s tau rank correlation analysis. This algorithm has been implemented in a software called “FLAVER” (Huang *et al.* 2024, Yao *et al.* 2024). The FLAVER algorithm was proposed to identify the key transcription factors by correlating their target’s binding potential with target’s extent of differential expression.

Four software packages, SCENIC, VIPER, Dorothea, and ChEA3, were recently developed to tackle the problem of identifying key TFs from transcriptome data using specific subroutines. SCENIC predicts genomic enhancers along with candidate upstream TFs and links these enhancers to candidate target genes (Aibar *et al.* 2017, Bravo González-Blas *et al.* 2023). The VIPER algorithm inference protein’s activity from expression profiling data uses the genes that are most directly regulated by a given protein as an accurate reporter of its activity (Alvarez *et al.* 2016). Dorothea identifies transcription factor activity enhance markers in transcriptome datasets using signed TF-target gene interactions (Garcia-Alonso *et al.* 2019). ChEA3 is a transcription factor enrichment analysis tool that ranks TFs associated with user gene list using background database including TF–gene co-expression from RNA-seq studies, TF–target associations from ChIP-seq experiments, and TF–gene co-occurrence computed from crowd-submitted gene lists (Keenan *et al.* 2019).

The present study proposes a method to construct directed TF-target sets, which are ranked by the artificial neural network (ANN) score. These ranked sets serve as a required input for the TransPilot server. TFs exhibit diverse regulatory characteristics toward target genes, promoting transcription in some cases but inhibiting it in others. Modeling the directions of TF to target regulation remains challenging, primarily due to: (i) lack of directional information in currently available TF-target datasets; and (ii) the fact that current TF discovery algorithms struggle to effectively utilizing the positive and negative regulatory information for genes in TF-target datasets when performing enrichment analysis. We determined the directions (positive or negative) for the regulatory relationships between TFs and their target genes by calculating the correlation between their expression levels in a reference transcriptome dataset. We also applied a correlation-based algorithm to test whether the ranked and direction-annotated TF-target gene sets were significantly enriched in the ranked DEGs.

It is important to note that the FLAVER software package exclusively considers genes which contain TFBSs for the TFs of interest. However, such genes constitute only a limited proportion of the entire DEG list. For genes lacking TFBSs and thus lacking LLR values, gene expression level measures are still available, and they may exhibit distinct expression patterns compared to genes possessing TFBSs. This non-target gene population can serve as a crucial background set, significantly improving the specificity and robustness of the correlation statistics. In this study, an LLR imputation was applied to the genes lacking TFBSs to fill in the missing LLR values. The TransPilot server, as outlined in this article, incorporates theoretical enhancements and software upgrades in these respects.

## 2 Methods

### 2.1 System description

**Figure 1:**
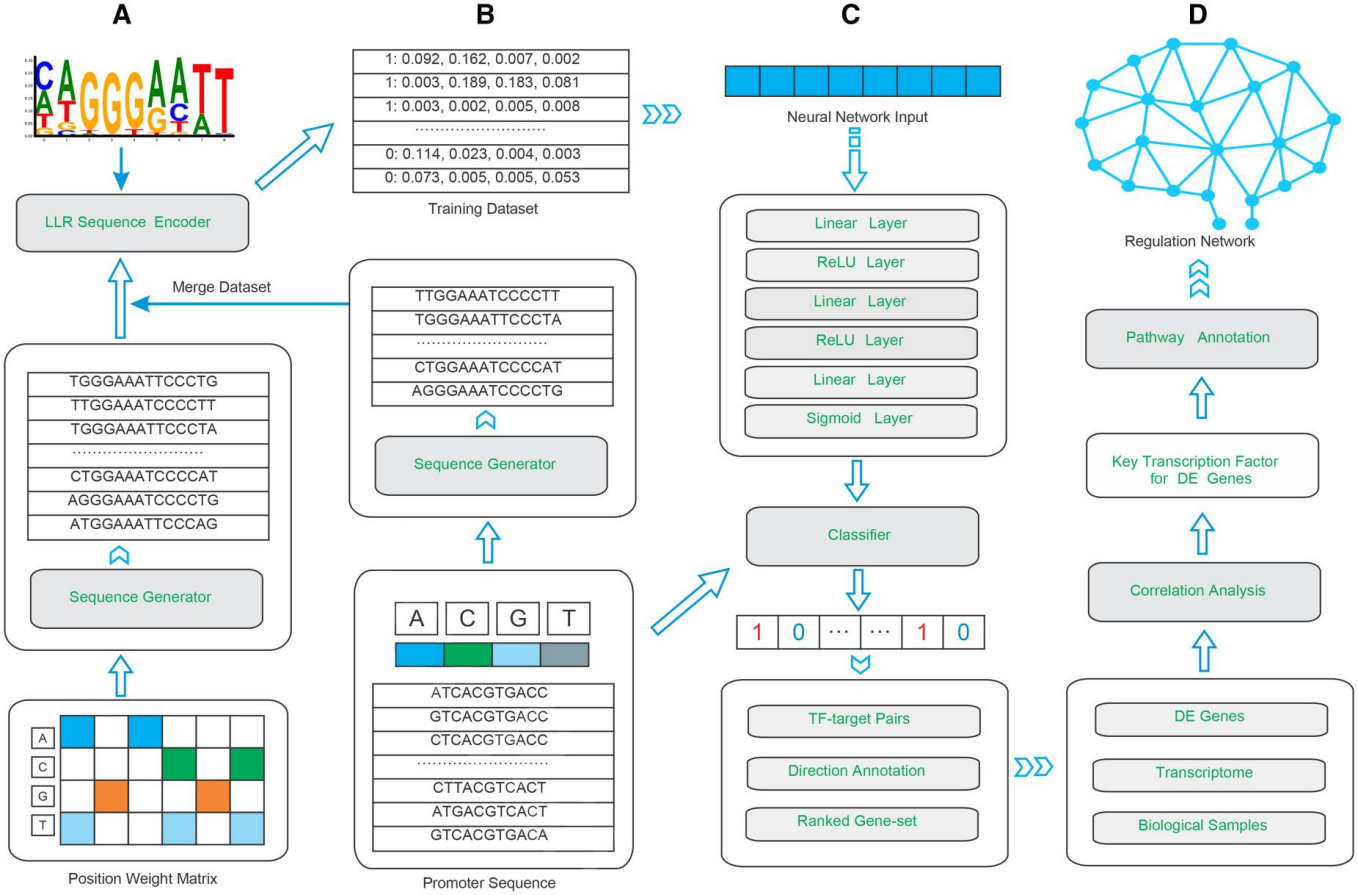
The schema of TransPilot server. (A) LLR-matrix coding for positive training nucleotide sequences. (B) LLR-matrix coding for negative training nucleotide sequences. (C) The ANN model and creation of the ranked and directed TF-target dataset. (D) Creation of ranked gene list from transcriptome data, identification of key TFs by TransPilot, and finally build the regulation network.

This study focuses on the identification of key TFs from transcriptome data and the project can be divided into two subproblems: (i) how to precisely locate the TFBSs in the promoter sequence (1 kb up-TSS + 100 bp down-TSS) and then build the TF-target gene pairs; and (ii) how to accurately discover the key TFs regulating the transcription levels of the DEGs among biological samples. The schema of TransPilot is demonstrated in Fig. 1, which includes subroutine of constructing positive and negative datasets (Fig. 1A and B), training ANN models then creating ranked and directed TF-target sets (Fig. 1C), building ranked DEG lists then identifying key TFs and creating regulation networks (Fig. 1D).

### 2.2 Algorithm for locating the TFBSs in the promoter sequence

The solution to problem (i) relies on the position weight matrix (PWM) of TF’s motif, as determined by methods such as ChIP-seq. This matrix, with dimensions of four rows by N columns (where N is the motif length), describes the probability of each of the four bases appearing at each position within the motif, representing a generalized sequence pattern. TFs possess specific DNA-binding domains that bind to motifs with specific sequence patterns and thereby function to activate or repress gene transcription. The PWM provides a precise and measurable scoring standard for these generalized sequence patterns.

This study acquired a dataset of 2624 motif PWMs, which are recognized by 849 *Homo sapiens* TFs from the Hocomoco (Vorontsov *et al.* 2024) and Jaspar (Rauluseviciute *et al.* 2024) databases. Positive training datasets were generated using a random procedure with nucleotide drawings according to probabilities derived from the PWMs. Let *i*-nucleotide base set b∈{A, T, C, G}, sequence to be generated as *S* = [*s*_1_, *s*_2_, *s*_3_, …, *s_k_*], $p_{b}^{k}=(p_{A}^{k},{\;p}_{C}^{k},{\;p}_{G}^{k},{\;p}_{T}^{k})$ is the probability of nucleotide *b* at the *k*th position and satisfies $\sum p_{b}^{k}=1,\;p_{b}^{k}\geq 0$. First, generate *i* uniformly distributed random numbers $u_{i}∼U(0,1)$, then *s_k_* can be specified in Equation (1). The negative training sequences were generated by a similar approach using nucleotide frequencies of promoter background sequences, with each position using the same probabilities of nucleotide draws according to $p_{b}=(p_{A},p_{C},p_{G},p_{T})$. Detailed data of the positive and negative training sequence are provided in Files 1 and 2, available as supplementary data at *Bioinformatics Advances* online.


(1) $$s_{k}=b_{i}\;\mathrm{if}\;\sum_{i<b}{p_{i}}^{k}\leq u_{i}\leq \sum_{i\leq b}{p_{i}}^{k}$$

The traditional method “slides” the PWM along a promoter DNA sequence, calculates the average LLR at each corresponding position, and uses algorithms such as dynamic programming (FIMO) to calculate the probability that the average LLR of the background model is greater than the observed value, thereby predicting whether the sequence at a position is a potential TFBS. The LLR quantifies the probability that a nucleotide base pair in the promoter sequence matches a base pair pattern in the motif’s position weight matrix (PWM) of a TF, however, in the process of calculating the *P*-value using dynamic programming, we lost the numerical differences between individual LLR values and the information regarding their relative positions. Deep learning methods have, in this aspect, compensated for the limitations of dynamic programming algorithms. Let sequence *S* = [*s*_1_, *s*_2_, …, *s*_3_, …, *s_i_*] with length *l*. Commonly used feature matrix *X* = [*x*_11_, *x*_12_, *x*_13_, *x*_14_, …, *x*_i1_, *x*_i2_, *x*_i3_, *x*_i4_] is constructed by one-hot encoding, where each base pair *S_i_*(A, C, G, and T) is denoted as the one-hot vector [*x_i_*_1_, *x_i_*_2_, *x_i_*_3_, *x_i_*_4_], more specifically [1, 0, 0, 0]A, [0, 1, 0, 0]C, [0, 0, 1, 0]G, and [0, 0, 0, 1]T, respectively. In this study, a log likelihood ratio (LLR) feature matrix was developed as *Z* = [*z*_1_, *z*_2_, *z*_3_, …, *z_k_*], and *z_k_* has the form of Equation (2).


(2) $$z_{k}=\text{ln}\frac{q(k,\;L_{k})}{p(L_{k})};\;1\leq k\leq w$$

where “*w*” represents the width of the motif, “*L*” denotes the location being considered, *L_k_* is the nucleotide at position “*k*” within this location, *p*(*L_k_*) is the background probability of observing nucleotide *L_k_* estimated from the frequency of *L_k_* in that sequence, and *q*(*k, L_k_*) is the probability of observing nucleotide *L_k_* estimated from the frequency of the *k*th location in the motif. The LLR feature matrix for a motif present in a sequence of width “*w*” is constructed as all LLRs taken over all locations of “*k*,” where “*w*” is defined as the number of locations in the motif. The LLR feature matrix is used as input to train a three-layer ANN. The calculation process of these layers is described by Equation (3):


(3) $$Y_{\mathrm{ANN}}={\mathrm{Sigm}}_{1}(w_{32}^{3}\cdot ({\mathrm{Relu}}_{32}(w_{64}^{2}\cdot ({\mathrm{Relu}}_{64}(w_{k}^{1}\cdot z_{k}+b_{k}^{1})+b_{64}^{2})+b_{32}^{3})$$

The *w_x_* and *b_x_* are weight matrix and bias vector of the linear function in each layer, respectively. The Sigm_*x*_ and ReLu_*x*_ are the Sigmoid and ReLu function, respectively. The subscript “*x*” represents the dimension of the input parameter. The “·” is an elementwise product operator. *Y*_ANN_ is the output of the ANN model, which is followed by a classifier for PWM binding instances. The parameters used for training the model are learning_rate = 5E-4, epochs = 100, weight_decay = 1E-4, batch_size = 512, train: test = 7:3. The above algorithm has been implemented in a Python extension package named PyTFBS (v1.0.5), which is available under a free academic license.

The TF-target sets, containing TF-target pairs, were constructed according to the genes targeted by a specific TF derived from the TFBS candidates identified by the ANN classifier. The TF-target pairs were ranked by the ANN score (*Y*_ANN_, > 0.5) and the directions (positive or negative) of the regulatory relationships for each pair were determined by the correlation coefficient between their expression levels in a reference transcriptome dataset obtained from the Human Protein Atlas database (Uhlén *et al.* 2015).

### 2.3 Discover the key TFs regulating the transcription level of the DEGs

A hybrid strategy was employed by the TransPilot server to solve problem (II). TransPilot constructs the TF-target set, in which the genes were categorized into two groups: (I) genes that have PWM binding instances in their promoter sequence, and (II) genes lacking PWM binding sites in their promoter sequence. For the genes in group (I), the LLR values of the binding site are available and can be transformed into the gene’s rank values by the ANN model in the TF-target set. For the genes in group (II), although the LLR values of their binding sites are unknown, the expression levels of these genes are available, and therefore must be incorporated into the calculation. The adopted strategy was to populate the genes in group (II) with random LLR values, where the random values are drawn from the range [0, min(Rank_*i*_)].

Employing the same strategy developed previously (Yao *et al.* 2024), the present study identified key transcription factors from transcriptome data by testing the significance of the correlation between the order of genes in the TF-target set and the corresponding order in the DEG list. The analysis emphasizes genes with large weights and de-emphasizes genes with small weights. The consistency of ranks between genes in the TF-target set and DEG list can be measured by a weighted rank correlation statistical method. Let *S_i_* and *L_i_*, *i *= 1, …, *n* be the ranks of the genes in the TF-target set and the DEG list, respectively. In addition, let (*i*, *R_i_*), *i *= 1, …, *n* be the pair-wise ranks, where *R_i_* is the rank in “*L*” corresponding to gene whose rank in *S_j_* is *i*, *j *= 1, …, *n*. As found by Shieh (1998), the weighted Kendall’s τ has the form of Equation (4). The limiting distribution (LD) can be derived from Equation (5). As the value of “*n*” approaches infinity, the LD value approaches *N*(0, 1), and thus, the *P*-value can be estimated.


(4) $$\tau_{w}=2/\left[{\left(\sum_{i}^{n}v_{i}\right)}^{2}-\sum_{i}^{n}v_{i}^{2}\right]\cdot \sum_{i>j}^{n}v_{i}v_{j}\text{sgn}(i-j)\text{sgn}(R_{i}-R_{j})$$

If *X *< 0, = 0, or > 0, then sgn (*X*) = −1, 0, or 1. The *v_i_* denotes the weighting function which is specified in Equation (6), where *R_i_* are ranks of genes in the TF-target set or the DEG list, the index “*i*” is bounded by [0, *n*] and function values range from 0 to 1.


(5) $$\text{LD}=\sqrt{n}\tau_{w}\frac{3\underset{n\to \infty }{\text{lim}}n^{-1}\sum_{x}^{n}v_{x}}{2\sqrt{\underset{n\to \infty }{\text{lim}}n^{-1}\sum_{x}^{n}v_{x}^{2}}}$$

(6) $$v_{i}=(1-\frac{\left|R_{i}\right|}{\text{max}\left\{\left|R_{i}\right|\right\}})$$

The genes in group (II) are assigned a uniform weighting value of 0.25, and the genes in group (I) are assigned weighting values ranging from 0.5 to 1.0 according to the linear transforming method. The weighting function *v_x_* employed in the test is delineated in Equation (7), where “*x*” ranges from 1 to *n*. The *R_xs_* and *R_xl_* are ranks of genes in the TF-target set and the DEG list, respectively. This weighting function operates under the assumption that the expression levels of the majority of genes in the genome remain constant, with differentially expressed genes accounting for a small proportion of the total number of genes. This function presupposes that the ANN scores of the majority of genes in the genome remain low. As indicated by Equation (6), the genes that exhibit a high extent of differential expression or possess high ANN scores will be assigned a higher weighting value.


(7) $$v_{x}=\frac{{\left[\left(1-\frac{\left|R_{\text{xs}}\right|}{\text{max}\left\{\left|R_{\text{xs}}\right|\right\}})\cdot (1-\frac{\left|R_{\text{xl}}\right|}{\text{max}\left\{\left|R_{\text{xl}}\right|\right\}}\right)\right]}^{0.5}+1.0}{2.0}$$

Utilizing these strategies, we have developed TransPilot server based on the schema proposed in Fig. 1. The server takes a ranked and directed TF-target set and a ranked DEG list as its input. Running the server with the data in the browser window will produce a result table containing the statistical results of the TF-target sets. There are five major steps built into the program: (i) matching the genes between the TF-target sets and DEG list; (ii) sorting genes first by ranks of the TF-target sets and then by the ranks of the DEG list; (iii) calculating the tau statistics for each TF-target set using Equation (4); (iv) calculating the *P*-value for the significance of each TF-target set using Equation (5); and (v) performing multiple testing correction for all *P*-value using the FDR method. The source code is available under an academic free license.

### 2.4 Evaluate TransPilot server using real-world and synthetic datasets

The performance of the new server was evaluated using a transcriptome dataset derived from a macrophage polarization experiment (Migliaccio *et al.* 2024). The genes in the DEG list were ranked by a score detailed in Equation (8).


(8) RANK=-Log10(FDR)·LogFC

The LogFC represents the log fold change for gene transcription levels between M1 samples and controls samples, and FDR denotes the corrected *P*-value. The DEG list was then analyzed using the TransPilot server and TF-target sets describing TFBSs. To assess the results, the target TFs playing roles in M1 polarization were selected based on recent literature reviews (File 3, available as supplementary data at *Bioinformatics Advances* online). For each target TF implicated in M1 polarization, we identified the *P*-value and rank of its corresponding TF-target set group in the prediction results. The RE score was utilized to evaluate the quality of the results according to Equation (9), where Rank_*i*_ is the rank of target TF *i*, “*n*” is the number of target TFs, and “*N*” is the total number of TFs. Finally, the ranks of the target TFs among four competitor servers were compared using the one side (greater) and paired *Z*-test statistical method.


(9) $$\mathrm{RE}=\frac{1}{n}[\sum_{i=1}^{n}-\mathrm{Log}10({\mathrm{Rank}}_{i}/N)]$$

Five transcriptome datasets, the heart, liver, spleen, lung, and kidney, were extracted from tissue profiling experiments deposited in GTEx database (Melé *et al.* 2015) to further evaluate the new server. The DEG list was ranked using a previously proposed ED score (Huang *et al.* 2024), detailed in Equation (10).


(10) $$\mathrm{ED}=x_{i}-[\left(\sum_{i=1}^{n}x_{i})\cdot (s_{i}/\sum_{i=1}^{n}s_{i}\right)]$$

The *x_i_* represents the expression level of gene *x* in sample *i*, s_*i*_ summarizes the expression levels of all genes in sample *i*, and “*n*” denotes the number of samples. The results were assessed through a literature survey of key TFs playing roles in these tissues.

The synthetic test DEG lists were generated as follows: (i) Twenty transcription factors were randomly selected from the TF-target set, and subsets of their target genes were generated by random sampling at a specified proportion (0.35–0.8); (ii) Three DEG lists correlated with the TF-target subsets were then generated based on correlation coefficients *R*, *R*2/3, and *R*1/3, respectively (*R* = 0.35–0.8); (iii) A completely random DEG list was generated and then evenly divided into three parts according to the rank of differential expression values. The genes from the three correlated DEG lists *R*, *R*2/3, and *R*1/3 were then injected into the top, middle, and bottom regions of the random DEG list, respectively, to produce the final synthetic DEG list. The workflow for generating the synthetic DEG lists was illustrated in Fig. 4B, and the testing results are available in File 4, available as supplementary data at *Bioinformatics Advances* online.

## 3 Results

### 3.1 ANN model training and system testing

**Figure 2:**
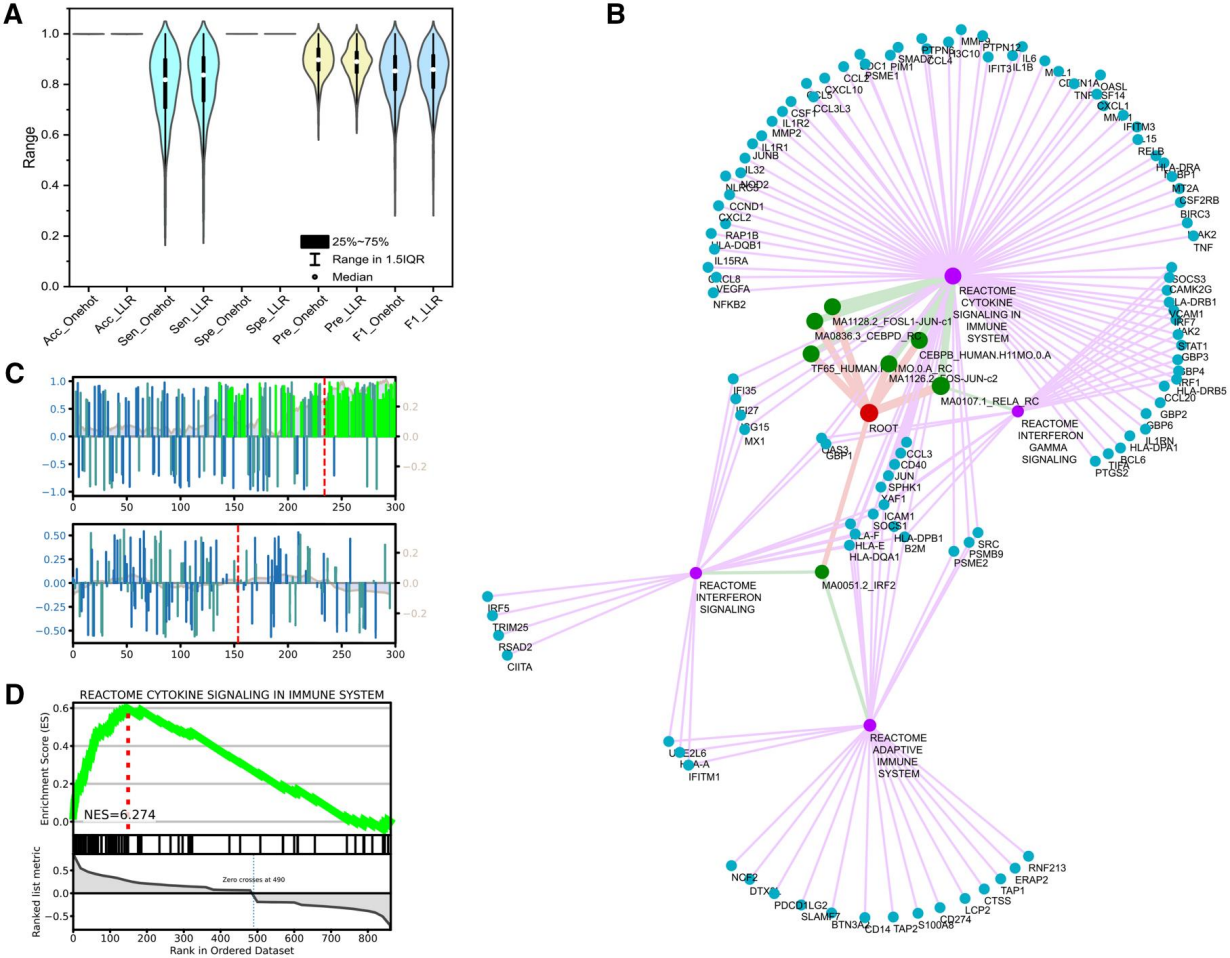
Result of ANN model training and system testing. The performance of the ANN models trained with one-hot and LLR array coding methods is demonstrated in plot A with a boxplot. Representative transcription regulation network for key TFs in M1 macrophages is illustrated in graph B. The correlation graphs for TF65, illustrating the relationship between its binding potential and the extent of differential expression of its target genes, are shown in graph C. The upper plot of C illustrates the genes that have a TF binding site, while the lower plot depicts genes lacking TF binding sites. The x-axis indicates the rank order of their differential expression levels. Bar height serves as a quantitative measure of the binding potential between a TF and its target genes. Bars rendered in dark green indicate the target gene is upregulated, while bars rendered in dark blue indicate downregulation. The filled curve in plot demonstrates the kernel density of bars. The red line indicates the weighted average of the bars. Bars highlighted in green represent the edge genes identified in the pathway enrichment analysis illustrated in plot D.

Two sets of ANN models were trained, one using one-hot encoding and the other using LLR array encoding. The results showed that the models based on the LLR array achieved an average accuracy of 99.9%, with an average sensitivity of 90%, specificity of 99.9%, precision of 88.2%, and F1 score of 84.1%. No significant difference was observed between the performance of the models trained using one-hot encoding and LLR array encoding method (Fig. 2A). Since the LLR array-based model has only one-quarter the parameters of the one-hot model, which theoretically lowers the risk of overfitting, the LLR array-based results were employed in all subsequent experiments. Based on the TFBSs predicted by the ANN model, three TF-target sets were constructed, with the ranking scores defined as follows: ANN score (ANN set), *sign*(correlation coefficient) × ANN score (Dir set), and correlation coefficient (CRank set).

Based on the transcriptome dataset from macrophage polarization experiments (Migliaccio *et al.* 2024), we generated a ranked DEG list for M1 cells versus control cells. The dataset contained 3,079 genes (FDR < 0.01, FC > 3.0), of which 1,508 were downregulated and 1,571 were upregulated. According to the TransPilot server analysis results, among the three TF-target sets—ANN, Dir, and CRank—49, 1,045, and 1,187 motifs reached statistical significance corresponding to 38, 448, and 503 TFs (FDR < 0.05), respectively. Fig. 2B presents a transcriptional regulatory network constructed from seven transcription factors: TF65, RELA, FOS-JUN, CEBPB, CEBPD, FOSL1-JUN, and IRF2. Among these, the transcriptional level difference of TF65-targeted genes in M1 macrophages were positively correlated with the binding potential of TF65 binding sites (Fig. 2C). Functional annotation revealed that these TF65-targeted genes were enriched in cytokine pathway genes (Fig. 2D).

### 3.2 Performance assessment of the TransPilot server

**Figure 3:**
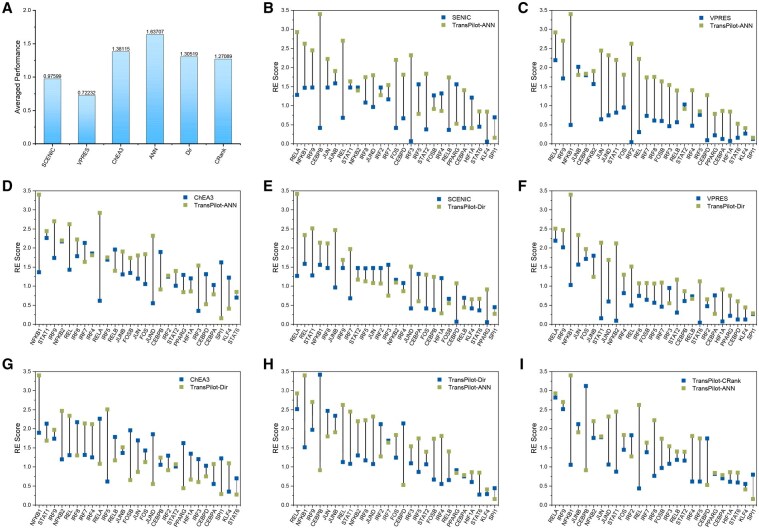
Performance of the TransPilot server and its competitors. The averaged performance (RE score) for TransPilot server and its competitors is illustrated in bar graph A. Pairwise comparisons for the RE scores of target TFs in results of TransPilot server and its competitors are shown in plot B to I.

A total of 27 macrophage polarization TFs were selected through literature reviews to assess the results. The prediction results of three competitor servers were compared with TransPilot server’s results. The results demonstrated that the average performance for TransPilot server with ANN TF-target set was highest, followed by ChEA3, Dir set, and CRank set (Fig. 3A). Pairwise comparison of the ANN set results with its competitors showed that the ANN set outperformed SENIC, VPRES, and ChEA3 for 19:8, 25:2, and 17:10 of TFs, respectively (Fig. 3B–D). Pairwise comparison of the Dir set results with its competitors showed that the ANN set outperformed SENIC, VPRES, and ChEA3 for 15:12, 23:4, and 12:15 of TFs, respectively (Fig. 3E–G). Pairwise comparison of the ANN set result with Dir and CRank set result demonstrated that the ANN set outperformed Dir and CRank set for 19:8 and 21:6 of TFs, respectively (Fig. 3H–I). The full results of TransPilot server and its three competitors are provided in File 5, available as supplementary data at *Bioinformatics Advances* online. It should be mentioned that, by incorporating the directions of TF-target regulation into the statistics, the *P*-value decreased by a factor of 10^9^ in average.

**Figure 4:**
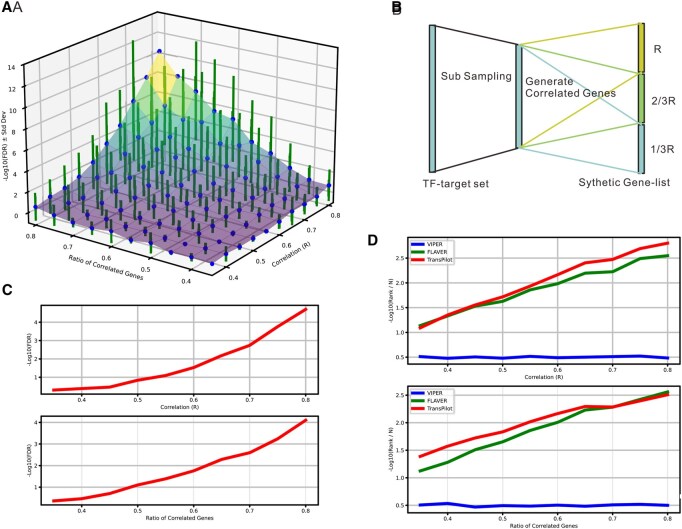
Performance of the TransPilot server on synthetic DEG list. Plot B illustrates the workflow for generating the synthetic DEG lists. Plot A shows the testing results for synthetic DEG lists generated with different *R* values (the correlation coefficient, x-axis) and ratio values (proportion of correlated genes, y-axis). The height of the points on the surface (z-axis) represents −log_10_(FDR), and the green bars indicate the mean ± standard deviation for 20 randomly selected transcription factors. Plot C presents the dynamics of −log_10_(FDR) with different *R* values (top panel) and ratio values (bottom panel). Plot D shows the dynamics of −log_10_(Rank/*N*) with different *R* values (top panel) and ratio values (bottom panel), where rank denotes the rank of the tested transcription factor in the prediction results, and *N* represents the total number of transcription factors.

The results of testing the TransPilot server using a completely random DEG lists showed that none of the candidate transcription factors reached statistical significance, with all FDR values exceeding 0.05. For the synthetic DEG lists, the results indicated that when the *R* value (simulating the correlation between target gene’s expression differences and transcription factor binding potential) and the ratio value (representing the proportion of correlated target genes) were below approximately 0.35, the FDR values calculated by TransPilot were higher than 0.05 (Fig. 4A). Moreover, the −log10(FDR) value increased progressively with increasing *R* values (top panel of Fig. 4C) and Ratio values (bottom panel of Fig. 4C), suggesting that the FDR calculated by the TransPilot algorithm exhibits a strong monotonic response to both *R* and ratio in the synthetic DEG lists. Because the ChEA3 and SCENIC packages do not directly support the ranks in the above synthetic DEG lists, similar analyses were therefore not performed using these tools. Although VPRES can accept ranked DEG lists as input, the −log10(rank/*N*) output by VPRES showed no clear pattern with the changes in *R* values (top panel of Fig. 4D) or ratio values (bottom panel of Fig. 4D). This discrepancy may be related to the specific way in which VPRES processes the rank values in the synthetic DEG lists.

### 3.3 Identification of tissue-specific TFs by TransPilot server

**Figure 5:**
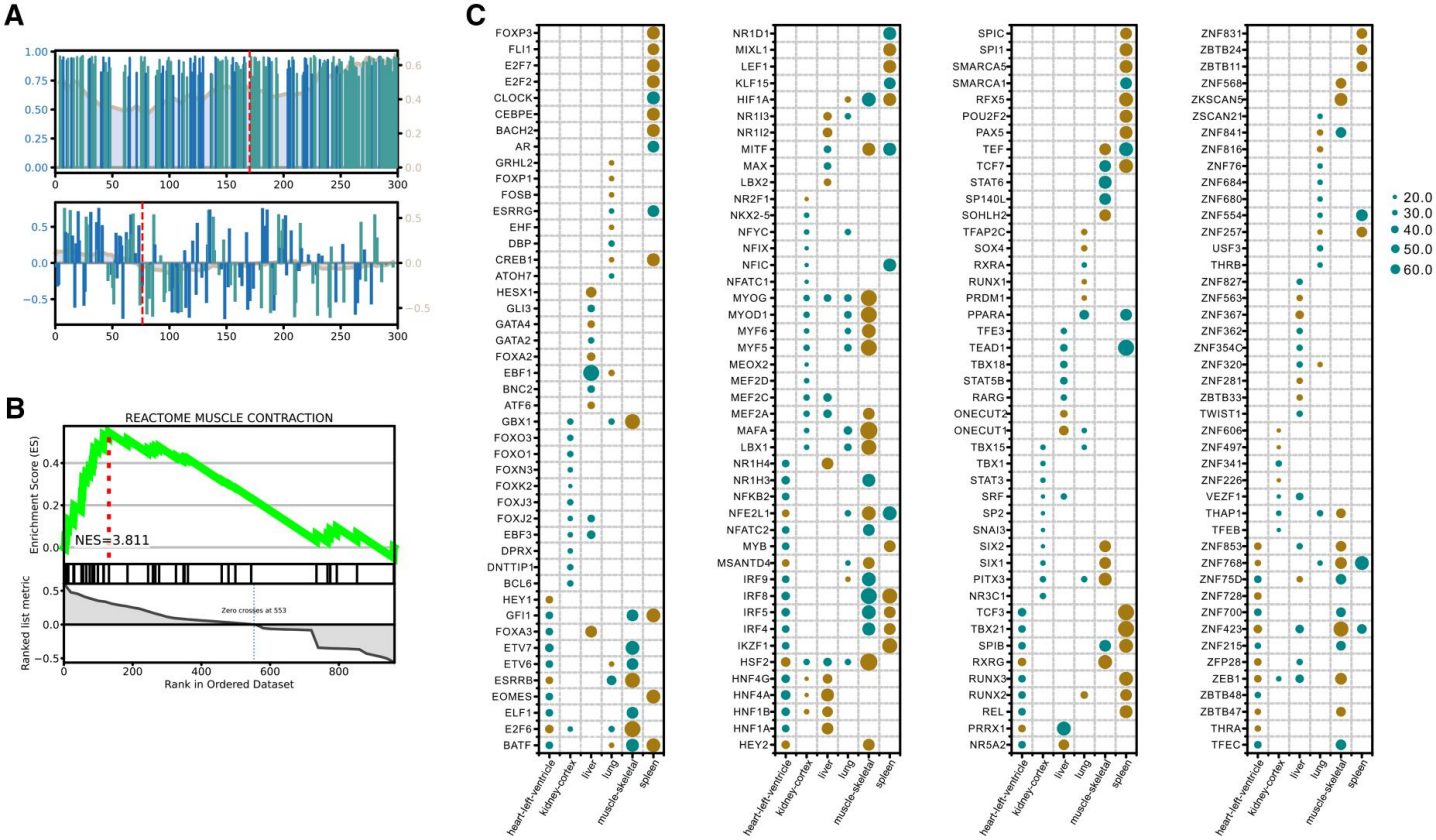
Top 30 key TFs for tissue-specific genes identified by TransPilot server. Plot A and B illustrate correlation between the binding potential of a representative key TF (MYOG) and the extent of differential expression of its targets identified by TransPilot server. The bars and lines are illustrated using the same color schema as Fig. 2. Dots in plot C represent the −Log_10_(*P*value) values from TransPilot analysis results, with green indicating negative correlation between the binding potential and extent of differential expression for target genes regulated by the TF, and yellow indicating positive correlation.

Based on transcriptome data from heart, liver, spleen, lung, kidney, and muscle tissues (Melé *et al.* 2015), we constructed tissue-specific DEG lists containing 2987, 4159, 4056, 2862, 2368, and 4644 genes, respectively (abs(ED) > 1.0). TransPilot analysis using the Dir set identified 94, 67, 225, 28, 13, and 238 key transcription factors regulating these tissue-specific genes in the heart, liver, spleen, lung, kidney, and muscle, respectively (FDR < 1 × 10^−20^). The top 50 TFs for each tissue are illustrated in Fig. 5C, with the full list provided in File 6, available as supplementary data at *Bioinformatics Advances* online.

We annotated these TFs that are dominantly expressed in these tissues based on the literature. In the heart, the top TFs are ZNF423, HSF2, and E2F6. Variants in the ZNF423 gene are significantly associated with both diastolic and systolic blood pressure levels (Evangelou *et al.* 2018). HSF2 primarily participates in the regulation of embryonic cardiomyocyte development, protein homeostasis in the heart, as well as stress response modulation in cooperation with HSF1 (Benjamin and McMillan 1998). E2F6 mainly functions as a cell cycle repressor in the heart, contributing to maintaining the low proliferative homeostasis of mature cardiomyocytes (Movassagh *et al.* 2006).

In the liver, the top TFs are HNF4α, NR1H4, and FOXA3. HNF4α is a key regulatory factor that promotes liver development, maintains hepatic identity, and facilitates liver regeneration (Van Dender *et al.* 2026). NR1H4 acts as an important regulator of bile acid, lipid, and glucose metabolism in the liver, and maintains systemic metabolic homeostasis through the FXR-SHP-FGF15/19 axis (Kliewer and Mangelsdorf 2015). FOXA3 is an important hepatocyte nuclear factor that plays a regulatory role in early liver development, maintains liver metabolic homeostasis in adulthood, and rapidly initiates tissue repair upon liver injury (Li *et al.* 2019, 2023, 2025). In kidney tissue, the most significant transcription factor was HNF1B. HNF1B not only guides kidney development during the embryonic stage but also regulates key physiological functions in adulthood, including electrolyte balance and urine concentration (Clissold *et al.* 2015).In the spleen, the most prominent transcription factors were TCF3, TBX21, and IRF8. The function of TCF3 in the spleen is to regulate B lymphocyte development and homeostasis and its deficiency directly leads to a significant reduction in B cell numbers in the spleen (Bain *et al.* 1994). TBX21 regulates T and B cells, playing a central role in immune responses and serving as a key coordinator of adaptive immunity (Johnson *et al.* 2020). IRF8 plays roles in regulating the differentiation fate of myeloid and lymphoid cells in spleen and modulates the intensity of both innate and adaptive immune responses, making it a key transcription factor in anti-infective immunity (Tamura *et al.* 2015, Salem *et al.* 2020). In the lung, the top TFs are ZNF841, ZNF816, and RUNX2. The functions of ZNF841 and ZNF816 have not yet been fully elucidated, whereas RUNX2 is mainly involved in the regulation of pulmonary fibrosis and epithelial–mesenchymal transition of lung (Fang *et al.* 2025).

In addition to HSF2, top TFs identified by TransPilot in muscle include MAFA, ZNF423, MYF5, and MYOG. MAFA is expressed at low levels in the investigated samples, and the research on the function of ZNF423 is still limited. MYF5 is a master myogenic regulatory factor that specifies skeletal muscle lineage commitment and initiates myoblast determination during embryonic and adult muscle regeneration (Braun and Arnold 1996, Beauchamp *et al.* 2000). MYOG is a terminal differentiation transcription factor that drives myoblasts to exit the cell cycle and activate skeletal muscle structural gene expression, leading to mature myofiber formation (Edmondson and Olson 1989, Buckingham and Rigby 2014).

## 4 Discussion

The directed TF-target sets and DEG list each contain several thousand genes. However, only the end-ranked items are usually informative, consisting of only a small part of the whole list. A consolidated overall parameter which quantifies the degree of agreement of the rank positions of genes between the TF-target set and DEG list with emphasis on the end-ranked part is needed. The directed TF-target sets contain both positive regulated and negative regulated TF-target pairs. The DEG list also contains up-regulated genes and down-regulated genes. Traditional gene-set enrichment analysis methods for measuring one-sided rank shift are not effective for the analysis of directed and ranked TF-target set and DEG list. The weighted Kendall’s tau statistic, a flexible solution proposed in this study, places more emphasis on items ranked at both ends than those in the middle, by customizing the weighting function. The idea of the statistic is to identify key TF-target sets by correlating the rank order of the targets with the corresponding rank orders of genes in the DEG list. The significant TF-target sets correlated with the DEG list detected by the algorithm tend to be reliable evidence to infer that the TF candidates play roles in the biological process studied.

The weighted Kendall’s tau statistics require that the TF-target set and the DEG list have the same length therefore an imputation for the ANN scores for genes lacking TFBS is needed. The imputation method for LLR values may modify the correlation characteristics of group (II) genes and introduce an artificial bias. To circumvent this potential issue, we have adopted the following measures: (i) Utilizing uniformly distributed random numbers to populate the ANN scores. This approach ensures that, after sorting the values of the TF-target set and DEG list, if the DEG list and TF-target set are not correlated, the ranks of the genes in the TF-target set are randomly arranged. This phenomenon is illustrated by the correlation graph illustrated in the bottom panel of Figs. 2C and 5A; (ii) To circumvent the introduction of artificial bias due to the discrepancy in range size, we have opted for an interval of random numbers that is equivalent in range to the ANN scores for the group (I) gene, situated close to the minimum value of ANN score for these genes; (iii) The genes in the TF-target set are weighted utilizing a piecewise weighting function. For group (I) genes, the weighting values can be converted to values ranging from 0.5 to 1.0 through a linear transformation. However, for group (II) genes, the weighting value should be located within the range of 0 to 0.5. A reasonable estimate would be the midpoint between 0 and 0.5, namely 0.25, which would satisfy the idea of emphasizing (and thus assigning a higher weight) genes with high ANN scores.

Even though we only tested TransPilot server on a restricted number of transcriptome datasets, the results demonstrate that TransPilot server enhances the outcomes in two exemplary cases: (i) the extent of differential expression of group (II) genes (Fig. 5A, bottom panel) is considerably different from the expression levels of the group (I) genes (Fig. 5A, top panel); (ii) the extent of differential expression of the group (I) genes is significantly correlated with the binding potential of the key TF (Fig. 2C, top panel). For TFs identified by TransPilot which are regulatory candidates for DEGs, the corresponding TFs should also exhibit a differential activity, so as to undertake their transcriptional regulation roles. This can be a tendency of differential transcription levels among the samples analyzed. However, one should bear in mind that the differences of the transcript abundance are not the only state of activity change for a TF, but it is beyond the scope of the presented study to investigate possible exceptions to this rule.

For TransPilot users, we recommend submitting a sufficiently long DEG list, typically 2000–3000 genes, with clearly ranked levels of differential expression. We apply an imputation approach to fill the rank values for genes lacking TFBS, and therefore an ideal DEG list should include both genes with TFBS and those without, so that the imputation method has a sufficiently large gene pool for sampling. The TransPilot server provides three TF-target sets, ANN, Dir, and CRank. For an initial pilot analysis, the ANN set without direction can be used. If users are sensitive to regulatory directionality, they may choose the Dir set or the CRank set. The Dir set contains more genes than the CRank set, because during the construction of the CRank set, genes with correlation coefficients below 0.1 and *P*-value > 0.05 were removed. In addition, we advise users to carefully interpret the FDR values reported by TransPilot, as an overly liberal significance threshold may lead to an implausibly large number of significant transcription factors. The TransPilot results should be interpreted as a ranked list, not a binary classification. It should also be noted that TransPilot is based on computational prediction methods, and its output does not constitute biological validation. Any identified TF candidates should be independently confirmed experimentally by methods such as knockdown, knockout, or chromatin profiling, before drawing final biological conclusions.

## Supplementary Material

vbag145_Supplementary_Data

## Data Availability

The TransPilot server can accessed at http://www.thua45.cn/transpilot. The source code has been deposited in Science Data Bank as code (Tinghua and Min 2026). TransPilot v1.0. DOI: 10.57760/sciencedb.31,554 URL: https://cstr.cn/31253.11.sciencedb.31554). The PyTFBS software package can be installed via Python’s pip package manager.
